# A circulating cell population showing both M1 and M2 monocyte/macrophage surface markers characterizes systemic sclerosis patients with lung involvement

**DOI:** 10.1186/s12931-018-0891-z

**Published:** 2018-09-24

**Authors:** Amelia Chiara Trombetta, Stefano Soldano, Paola Contini, Veronica Tomatis, Barbara Ruaro, Sabrina Paolino, Renata Brizzolara, Paola Montagna, Alberto Sulli, Carmen Pizzorni, Vanessa Smith, Maurizio Cutolo

**Affiliations:** 1 Research Laboratory and Academic Division of Clinical Rheumatology, Department of Internal Medicine, University of Genova, Polyclinic San Martino Hospital, Genoa, Italy; 2 Clinical Immunology, Department of Internal Medicine, University of Genova, Genoa, Italy; 3 Department of Rheumatology, Ghent University Hospital, Ghent, Belgium; 4 Department of Internal Medicine, Ghent University, Ghent, Belgium

**Keywords:** Systemic sclerosis, Interstitial lung disease, Pulmonary artery hypertension, Monocyte/macrophage phenotype, M1, M2, Innate immunity, Lung CT scan, Pulmonary function tests, Flow cytometry, Anti-topoisomerase antibody

## Abstract

**Background:**

Systemic sclerosis (SSc) is a disorder characterized by immune system alterations, vasculopathy and fibrosis. SSc-related interstitial lung disease (ILD) represents a common and early complication, being the leading cause of mortality.

Monocytes/macrophages seem to have a key role in SSc-related ILD. Interestingly, the classically (M1) and alternatively (M2) activated monocyte/macrophage phenotype categorization is currently under revision.

Our aim was to evaluate if circulating monocyte/macrophage phenotype could be used as biomarker for lung involvement in SSc. To this purpose we developed a wide phenotype characterization of circulating monocyte/macrophage subsets in SSc patients and we evaluated possible relations with lung involvement parameter values.

**Methods:**

A single centre cross-sectional study was performed in fifty-five consecutive SSc patients, during the year 2017. All clinical and instrumental tests requested for SSc follow up and in particular, lung computed tomography (CT) scan, pulmonary function tests (PFTs), Doppler echocardiography with systolic pulmonary artery pressure (sPAP) measurement, blood pro-hormone of brain natriuretic peptide (pro-BNP) evaluation, were performed in each patient in a maximum one-month period. Flow cytometry characterization of circulating cells belonging to the monocyte/macrophage lineage was performed using specific M1 (CD80, CD86, TLR2 and TLR4) and M2 surface markers (CD204, CD163 and CD206). Non-parametric tests were used for statistical analysis.

**Results:**

A higher percentage of circulating CD204^+^CD163^+^CD206^+^TLR4^+^CD80^+^CD86^+^ and CD14^+^CD206^+^CD163^+^CD204^+^TLR4^+^CD80^+^CD86^+^ mixed M1/M2 monocyte/macrophage subsets, was identified to characterize patients affected by SSc-related ILD and higher systolic pulmonary artery pressure. Mixed M1/M2 monocyte/macrophage subset showed higher percentages in patients positive for anti-topoisomerase antibody, a known lung involvement predictor.

**Conclusions:**

The present study shows for the first time, through a wide flow cytometry surface marker analysis, that higher circulating mixed M1/M2 monocyte/macrophage cell percentages are associated with ILD, sPAP and anti-topoisomerase antibody positivity in SSc, opening the path for research on their possible role as pathogenic or biomarker elements for SSc lung involvement.

**Electronic supplementary material:**

The online version of this article (10.1186/s12931-018-0891-z) contains supplementary material, which is available to authorized users.

## Background

Systemic sclerosis (SSc) is a rare autoimmune disease, characterized by progressive microvascular damage and fibrosis, involving almost all organs of affected patients and predictable by several biomarkers [1].

Interstitial lung disease (ILD) is a common and early complication in SSc patients, and a certain degree of ILD, in the form of non-specific interstitial pneumonia (NSIP), has been shown in 78% of SSc lung biopsies. Notably, among possible organ involvements in SSc, ILD evolves to the worse prognosis, being the leading cause of mortality in SSc patients [2].

In addition, patients affected by SSc-associated ILD have a high risk to develop cardiopulmonary disease and pulmonary hypertension. After pulmonary hypertension development, severe impairments in both physical and emotional domains of health-related quality of life were demonstrated [3]. The 3-year death rate in SSc patients affected by pulmonary hypertension was calculated to be 44–64% [4, 5].

SSc-associated ILD was demonstrated to be early recognized by lung computed tomography (CT) scan. On the other hand, the wide range of pulmonary function test (PFT) normal values (80–120% of predicted) may determine its reduced sensitivity [6].

Several studies recently highlighted the genetic and epigenetic aberrations involved in the SSc pathogenesis [7, 8]. Importantly, major gene signatures related to phenotype, activation and migration of macrophages demonstrated to be relevant to the progressive pulmonary fibrosis, indicating macrophages as key players [9, 10].

Intriguingly, imbalance in macrophage phenotype features and macrophage activation have been lately considered essential for the development of inflammatory-autoimmune, fibrotic, infective and neoplastic disorders characterized by lung involvement [11–16]. Macrophages have been initially categorized as classically (M1) or alternatively activated (M2), mirroring T cells categories. M1 macrophages express specific phenotype markers, including toll-like receptors (i.e., TLR2 and TLR4) and the co-stimulatory molecules CD80 and CD86, and are involved in triggering intensive inflammation and tissue damage [17]. M2 macrophages primarily express the mannose receptor-1 (CD206) and macrophage scavenger receptors (CD204 and CD163), and they are associated with T helper (Th) 2 response, tissue repair and fibrosis [18, 19].

Recently, classifications based on a wider spectrum of phenotypes of which M1 and M2 subsets would constitute the two extremes have been described [20].

Moreover, it was observed that the majority of alveolar macrophages combine M1 and M2 features in steady state and that the mixed M1/M2 phenotype can be altered by HIV infection [21].

Interestingly, a recent preliminary study demonstrated higher percentages of circulating mixed M1/M2 monocytes/macrophages in SSc patients compared to healthy subjects (HSs) [22]. The aim of the present study was to effectuate a wide phenotype characterization of circulating monocytes/macrophages in consecutive SSc patients stratified according to the severity of lung and right heart involvement, through lung CT scan imaging, PFTs, pro-BNP blood values, and Doppler echocardiography.

## Methods

### Study design

As part of the regular follow up approved by international guidelines for SSc, all patients underwent clinical examination and instrumental exams over a period of time of up to one month. In particular, lung CT scan, PFT with diffusing capacity of the lungs for carbon monoxide (DLCO), Doppler echocardiography with systolic pulmonary artery pressure (sPAP) measurement, pro-hormone of brain natriuretic peptide (pro-BNP) blood values, were performed for lung-right heart involvement evaluation. The assumption of medications was also considered.

The study was approved by the Ethics Committee of Polyclinic San Martino Hospital, Genoa, Italy (protocol number: 273-reg-2015).

Throughout the manuscript, the investigated cells were defined as circulating “monocytes/macrophages”. This is because we wanted to use as much surface markers as possible, including those expressed from mature and polarized macrophages, to better restrict the investigation to monocytes and macrophages, and to study the mixed M1/M2 phenotype, independently from the cell maturation state. In fact, we did not want to exclude also a possible presence of circulating cells in later maturation stages. Therefore, we used different gating strategies, as reported in Additional file 1, including typical markers of immature and mature cells, as previously described [22].

### Participants

Fifty-five consecutive SSc patients (50 females and 5 males, mean age 63 ± 13 years), undergoing complete disease staging in a day hospital setting at the Rheumatology Division of Genoa University, were enrolled in the study after written informed consent. Among the enrolled SSc patients, 36 were characterized by a limited cutaneous (lcSSc) disease form and 19 were characterized by a diffused cutaneous (dcSSc) disease form. SSc diagnosis was done according to the American College of Rheumatology (ACR)/European League Against Rheumatism (EULAR) 2013 criteria [23, 24].

Data from blood samples derived from a population of 27 sex and gender matched HSs, analysed in a recent preliminary study, were applied here for comparison with SSc patients, for the most significant results [22].

### Lung and right heart involvement parameters

Lung CT scan, PFTs and sPAP, pro-BNP value measurements were performed in each patient in the same period of the other examinations scheduled for SSc follow-up. Results were interpreted by the same operator for each type of diagnostic test.

Afterwards, patients were stratified according to the presence or the absence of any interstitial involvement at lung CT scan (SSc-ILD group versus SSc No-ILD group, respectively). Therefore, patients were stratified also according to the presence or the absence of single CT scan abnormalities, characteristically described in SSc lung involvement: ground glass opacities (defined as an area of increased attenuation in the absence of architectural distortion) of lower lobes, ground glass opacities of upper lobes, peripheral septal thickening, apical fibrotic (architectural distortion with reticular intra-lobular interstitial thickening) changes, diffused fibrotic changes, traction bronchiectasis and bronchiolectasis (dilatation of the airways in the peripheral portion of the lung) and enlarged mediastinal nodes [25].

As regards PFT, forced vital capacity (FVC), DLCO and FVC/DLCO ratio values were reported and analyzed for each SSc patient. In agreement with previous studies, the FVC/DLCO ratio higher than 1.5 was considered suggestive for pulmonary vasculopathy in SSc patients [26].

### Flow cytometry

After enrolment, peripheral blood was collected in a lithium-heparin single tube from each SSc patient.

To identify monocyte/macrophage lineage surface markers CD14-APC-Vio770 and CD45-VioGreen antibodies were used. The characterization of M2 phenotype was performed using CD204-PE, CD163-PE-Vio770 and CD206-PeerCP-Vio700, whereas the M1 phenotype was investigated using CD80-APC, CD86-VioBlue, TLR2-PE-Vio615 and TLR4-VioBright-FITC antibodies. CD66b-FITC was used to identify and exclude granulocytes (Miltenyi Biothech, Bergisch Gladbach, Germany).

A total of 0.1 ml of peripheral blood was incubated with 10 μl of antibody for 15 min at room temperature, then erythrocytes were lysed and leucocytes post-fixed. Afterwards, the flow cytometry analysis was performed.

Three initial gating strategies were implemented to investigate circulating monocyte/macrophage phenotype over total leucocyte population and included in the Additional file 1. The first initial gating strategy evaluated the CD14^+^ cells over total leucocyte population. In this CD14^+^ cell population, circulating monocytes/macrophages showing an M2 phenotype were characterized based on the expression of CD204, CD163 and CD206. Therefore, a second initial gating strategy evaluated the CD204^+^ cells in the leucocyte population, excluding lymphocytes, CD66b^+^ granulocytes, doublets and cellular debris. In the CD204^+^ population, circulating cells co-expressing CD163 and CD206 were detected to characterize monocytes/macrophages showing an M2 phenotype. Cells positive for M2 phenotype markers (CD204, CD163, CD206) and M1 phenotype markers (TLR4, CD80 and CD86) were investigated to identify the presence of cells with a mixed M1/M2 phenotype, as recently reported [22]. Although lymphocytes and neutrophils are excluded in the initial gating strategy starting from CD204 ^+ ^cells, no specific dendritic cell markers were investigated to discriminate these cells and then they might be probably present in a limited percentage in the M1/M2 mixed population.

Finally, a third initial gating strategy was made up to detect monocytes/macrophages showing prominently M1 surface markers CD80, CD86, TLR2 and TLR4 [27].

Flow cytometric analysis was performed using a Navios Flow Cytometer and the Kaluza analysis software (Beckman Coulter, Milan, Italy), evaluating a total of 5 × 10^6^ cells and detecting more than 30 events in the smallest subset investigated, according to consensus guidelines on the minimal residual disease [28].

### Statistical analysis

Data were analyzed using IBM SPSS Statistics Version 21.0. (IBM Corp: Armonk, NY). Non-parametric tests were applied for statistical analysis and in particular Mann-Whitney U test was used for comparing data with an ordinal distribution between two independent groups. Kruskal-Wallis test was chosen to assess significantly different distributions of continuous dependent variables by a categorical independent variable with more than two groups. Finally, bivariate Pearson’s correlation was calculated to measure linear relationship between two variables with ordinal distribution. A *p*-value lower than 0.05 was considered as statistically significant. The results were expressed as median ± standard deviation (SD) and graphically represented through box and whisker plots.

## Results

Demographics and clinical parameters are summarized in Table 1.

**Table 1:** Demographic clinical and imaging data from the whole systemic sclerosis patient population

Demographic, clinical and imaging data in SSc PTs	Mean ± SD OR number-percentage
Age (years, mean ± SD)	63 ± 13
Sex (females/males)	50/5
RP duration (years, mean ± SD)	5.8 ± 10
SSc duration (years, mean ± SD)	8.4 ± 6
SSc form = LcSSc/dcSSc (*n* = %)	36 = 65.5% / 19 = 34.5%
ANA (*n* = %)	55 = 100%
ACA (*n* = %)	20 = 36.4%
Anti-Scl-70 Ab (*n* = %)	23 = 41.8%
ILD at CT scan (*n* = %)	37 = 67.3%
Ground glass opacities, lower lobes (*n* = %)	13 = 23.6%
Ground glass opacities, upper lobes (*n* = %)	8 = 14.5%
Ground glass opacities, upper and lower lobes (*n* = %)	8 = 14.5%
Peripheral septal thickening (*n* = %)	31 = 56.4%
Apical fibrotic changes (*n* = %)	20 = 36.4%
Diffused fibrotic changes (*n* = %)	15 = 27.3%
Enlarged mediastinal nodes (*n* = %)	16 = 29.1%
Traction bronchiectasis and bronchiolectasis (*n* = %)	16 = 29.1%
FVC% (mean ± SD)	104 ± 24
DLCO/VA% (mean ± SD)	71.5 ± 20
sPAP mmHg (mean ± SD)	34 ± 7
Pro-BNP (pg/ml, mean ± SD)	1423 ± 5119
On immunosuppressive therapy (*n* = %)	32 = 56.1%
On glucocorticoids (*n* = %)	9 = 16.4%
On ERAs (*n* = %)	16 = 28.1%

Data are expressed as means ±standard deviations or numbers = percentages of the total population. *SSc* Systemic sclerosis, *PTs* patients, *RP* Raynaud’s phenomenon, *SD* standard deviation, *ILD* interstitial lung disease, *ANA* Anti-nuclear antibody, *ACA*: Anti-centromere antibodies, *Ab anti-Scl70* anti-topoisomerase antibody, *CT* computed tomography, *FVC* forced vital capacity, *DLCO* diffusing capacity of the lungs for carbon monoxide, *sPAP* systolic pulmonary artery pressure, *pro-BNP* prohormone of brain natriuretic peptide, *ERAs* Endothelin 1 receptor antagonists. No other vasomodulating therapies were used by the selected SSc patients

Only 5 males were enrolled among the SSc patient population, consequently the sex variable was not used for the analysis.

### Associations between auto-antibody positivity, pro-BNP blood values, disease form, and monocyte/macrophage phenotype

**Fig. 1:**
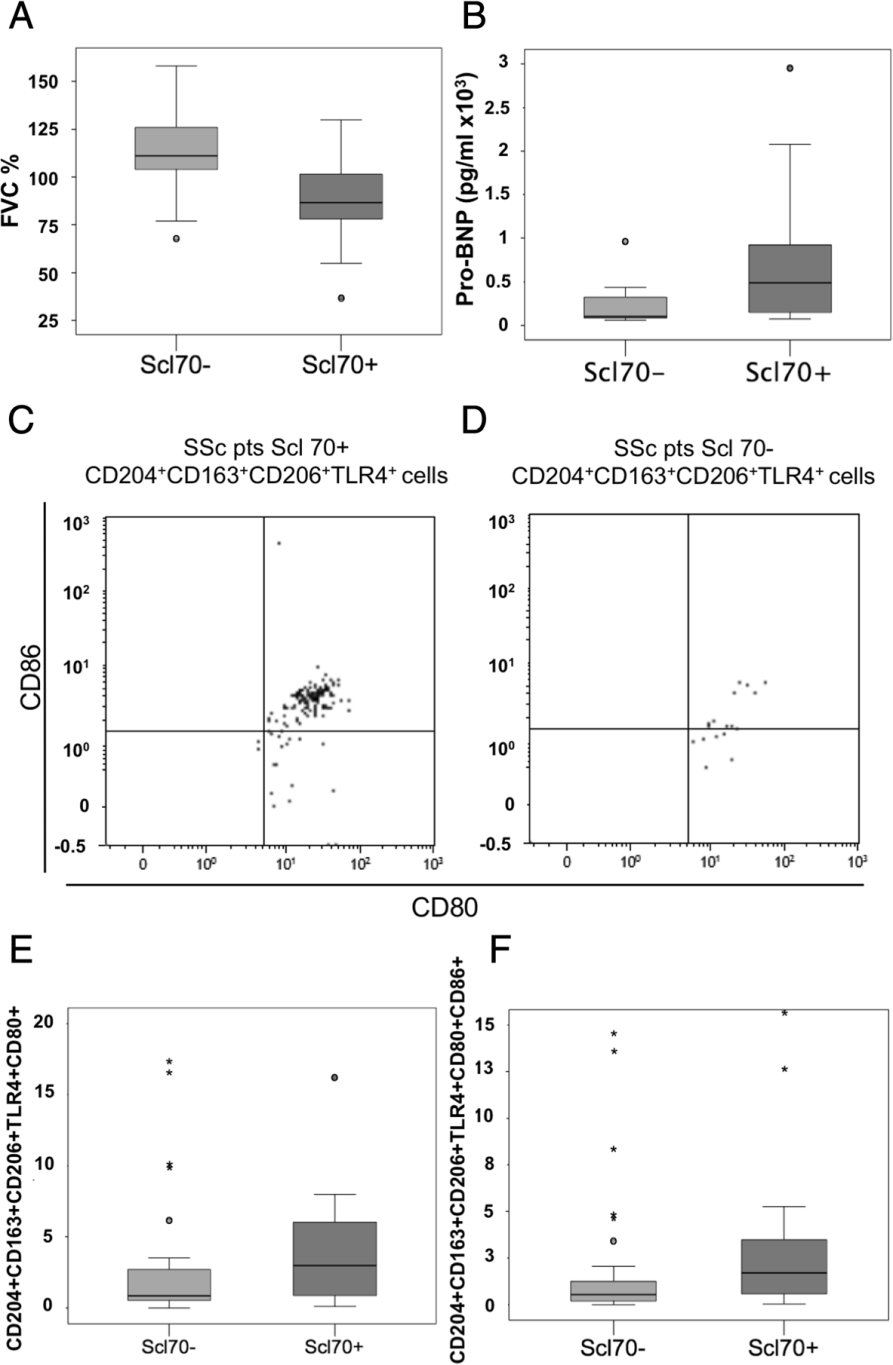
Ab anti Scl70 positivity: associations with FVC%, Pro-BNP blood values and mixed M1/M2 cells percentages. **a** and **b**, clinical associations of Anti-Scl70 Ab positivity with lower FVC% and higher pro-BNP values are shown. **c** and **d** show the representative dot plots from the flow cytometry analysis of the mixed M1/M2 CD204^+^CD163^+^CD206^+^TLR4^+^CD80^+^CD86^+^ cell subset is shown in patients with positive and negative Ab anti-Scl70. Significant differences (*p* = 0.027) are shown between average percentages of circulating mixed M1/M2 subset CD204^+^CD163^+^CD206^+^TLR4^+^CD80^+^ over total CD204^+^ cells, in Scl70+ vs Scl70- patients (**e**) and between percentage of circulating mixed M1/M2 subset CD204^+^CD163^+^CD206^+^TLR4^+^CD80^+^CD86^+^ over total CD204^+^ cells, in Scl70+ vs Scl70- patients (**f**). Anti-Scl70 = Anti-topoisomerase; FVC = forced vital capacity; pro-BNP = prohormone of brain natriuretic peptide

Anti-topoisomerase antibody (Anti-Scl70) positivity was associated with lower FVC% (Scl70 + = 84.5 ± 14% vs. Scl70- = 112.8 ± 22; *p* < 0.0001) and higher pro-BNP values (Scl70+ = 790 ± 883 vs. Scl 70- = 213 ± 243 *p* = 0.01) (Fig. 1a and b).

In the circulating CD204^+^ cell population of Scl70 positive SSc patients, by Mann-Whitney test, several mixed M1/M2 macrophage subsets showed higher percentages compared to Scl70 negative SSc patients: CD204^+^CD163^+^CD206^+^TLR4^+^CD14^−^ cells (Scl70+ = 2.4 ± 4.6%, vs. Scl70- = 0.64 ± 7.9%, *p* = 0.036), CD204^+^CD163^+^CD206^+^TLR4^+^CD80^+^ cells (Scl70+ = 8.2 ± 8.2% vs. Scl70- = 0.86 ± 4.4%, *p* = 0.027, Fig. 1e), CD204^+^CD163^+^CD206^+^TLR4^+^CD86^+^ cells (Scl70+ = 2.2 ± 6.9% vs. Scl70- = 0.7 ± 3.6%, *p* = 0.046), CD204^+^CD163^+^CD206^+^TLR4^+^CD80^+^CD86^+^ cells (Scl70+ = 1.6 ± 6.7% vs. Scl70- = 0.54 ± 3.6%, *p* = 0.036, Fig. 1c, d, f).

Using HS data, as reported in the Methods section, Kruskal-Wallis test was performed and significantly lower percentages for the same cell populations in HSs were obtained (HSs values for CD204^+^CD163^+^CD206^+^TLR4^+^CD14^−^ cells = 0.17 ± 0.41%, *p* < 0.0001 vs. SSc patients, pairwise comparison: HSs vs Scl70- *p* = 0.05, HSs vs Scl70+ *p* < 0.0001; CD204^+^CD163^+^CD206^+^TLR4^+^CD80^+^ cells = 0.16 ± 0.48%, *p* < 0.0001 vs. SSc patients, pairwise comparison: HSs vs Scl70- *p* = 0.001, HSs vs Scl70+ *p* < 0.0001; CD204^+^CD163^+^CD206^+^TLR4^+^CD86^+^ cells = 0.18 ± 0.36%, *p* < 0.0001 SSc patients, pairwise comparison: HSs vs Scl70- *p* = 0.001, HSs vs Scl70+ *p* < 0.0001; CD204^+^CD163^+^CD206^+^TLR4^+^CD80^+^CD86^+^ cells = 0.08 ± 0.29%, *p* < 0.0001 SSc patients, pairwise comparison: HSs vs Scl70- *p* = 0.001, HSs vs Scl70+ *p* < 0.0001, Additional file 2).

No association was reported between Scl70 Ab positivity and cells expressing exclusively M1 or M2 phenotype markers.

Anti-centromere antibodies (ACA) positivity was associated with older age at the time of the study (ACA+ = 70 ± 7 vs. ACA- = 57 ± 14 years, *p* = 0.002), longer SSc duration (ACA + = 10 ± 6 vs. ACA- = 7 ± 6 years, *p* = 0.049), higher FVC percentage (ACA+ = 118 ± 19% vs. ACA- = 94 ± 21%, *p* < 0.0001), and lower gammaglobulin percentage values (ACA+ = 14.8 ± 3% vs. ACA- = 17.3 ± 3%, *p* = 0.004).

ACA positive patients showed a higher percentage of CD14^+^ cells (ACA+ = 7.3 ± 1.9% vs. ACA- 6.3 ± 2.4%, *p* = 0.022). Several cells more clearly polarized towards an M1 or M2 phenotype segregated with ACA positivity: CD14^+^TLR2^+^ (ACA+ = 7.2 ± 2.6% vs. ACA- = 6 ± 3%, *p* = 0.046), CD14^+^CD163^+^ (ACA+ = 7 ± 1.9% vs. ACA- = 5.8 ± 2.6%, *p* = 0.025), and CD14^+^CD204^+^CD163^+^ (ACA+ = 0.18 ± 0.4% vs. ACA- = 0.08 ± 0.3%, *p* = 0.011).

Only CD204^+^CD163^+^CD206^+^TLR4^+^CD80^+^ cells, in the circulating CD204^+^ cells, showed higher percentages in dcSSc compared to lcSSc patients (dcSSc = 2.96 ± 8% vs. lcSSc = 0.94 ± 5%, *p* = 0.047). The Kruskal-Wallis test, executed adding HSs data, showed significantly lower CD204^+^CD163^+^CD206^+^TLR4^+^CD80^+^ cell percentages in comparison to both lcSSc and dcSSc (0.16 ± 0.48%, *p* < 0.0001, globally and after pairwise comparison).

### Associations between lung disease evaluated at CT scan and monocyte/macrophage phenotype

The only gating strategy that effectively highlighted differences in circulating monocyte/macrophage phenotype between SSc-ILD versus SSc-No ILD group was the one based on initial gating of CD204^+^ cells (Table 2).

**Table 2:** The CD204 positive cell population percentages are shown in patients with (SSc-ILD) or without interstitial lung disease (SSc-No ILD) at lung CT scan and healthy subjects (HSs)

Analysis of circulating CD204+ cells	SSc-ILD (37)	SSc-No-ILD (18)	p (MW)	HSs (27)	*p* (KW)
CD204^+^ (%)	0.5 ± 0.40	0.8 ± 0.7	*p* = 0.13	0.7 ± 0.3	0.21
CD204^+^CD163^+^ (%leukocytes)	0.08 ± 0.22	0.09 ± 0.14	*p* = 0.65	0.03 ± 0.03	**0.001**
CD204^+^CD163^+^ (%CD204^+^)	13.7 ± 15	8.4 ± 13	***p*** **= 0.034**	6.3 ± 3	**< 0.0001**
CD204^+^CD163^+^TLR4^+^ (%leukocytes)	0.03 ± 0.22	0.02 ± 0.15	*p* = 0.34	0.008 ± 0.01	**< 0.0001**
CD204^+^CD163^+^TLR4^+^ (%CD204^+^)	6.2 ± 16	2.9 ± 15	***p*** **= 0.025**	1.4 ± 1.7	**< 0.0001**
CD204^+^CD163^+^CD206^+^ (%leukocytes)	0.01 ± 0.1	0.01 ± 0.05	*p* = 0.65	0.008 ± 0.01	**0.001**
CD204^+^CD163^+^CD206^+^ (%CD204^+^)	4 ± 7.4	1.9 ± 5.6	*p* = 0.07	1.1 ± 1.2	**< 0.0001**
CD204^+^CD163^+^CD206^+^TLR4^+^ (%leukocytes)	0.014 ± 0.1	0.11 ± 0.05	*p* = 0.20	0.003 ± 0.004	**< 0.0001**
CD204^+^CD163^+^CD206^+^TLR4^+^ (%CD204^+^)	2.7 ± 7.3	1.1 ± 5.9	***p*** **= 0.013**	0.5 ± 0.6	**< 0.0001**
CD204^+^CD163^+^CD206^+^ TLR4^+^CD14^+^(%leukocytes)	0.003 ± 0.014	0.004 ± 0.003	*p* = 0.795	0.001 ± 0.003	**0.008**
CD204^+^CD163^+^CD206^+^ TLR4^+^CD14^+^(%CD204^+^)	0.73 ± 1.4	0.27 ± 0.54	*p* = 0.097	0.20 ± 0.38	**< 0.0001**
CD204^+^CD163^+^CD206^+^ TLR4^+^CD14^−^ (%leukocytes)	0.009 ± 0.08	0.006 ± 0.05	*p* = 0.092	0.001 ± 0.002	**< 0.0001**
CD204^+^CD163^+^CD206^+^ TLR4^+^CD14^−^ (%CD204+)	1.93 ± 6.56	0.6 ± 5.69	***p*** **= 0.029**	0.17 ± 0.41	**< 0.0001**
CD204^+^CD163^+^CD206^+^ TLR4^+^CD80^+^ (%leukocytes)	0.01 ± 0.09	0.004 ± 0.05	***p*** **= 0.041**	0.001 ± 0.003	**< 0.0001**
CD204^+^CD163^+^CD206^+^ TLR4^+^CD80^+^ (%CD204^+^)	2.07 ± 6.83	0.5 ± 5.33	***p*** **= 0.010**	0.16 ± 0.48	**< 0.0001**
CD204^+^CD163^+^CD206^+^ TLR4^+^CD86^+^ (%leukocytes)	0.008 ± 0.08	0.005 ± 0.04	*p* = 0.082	0.001 ± 0.002	**< 0.0001**
CD204^+^CD163^+^CD206^+^TLR4^+^ CD86^+^ (%CD204^+^)	1.16 ± 5.8	0.72 ± 4.1	***p*** **= 0.023**	0.19 ± 0.36	**< 0.0001**
CD204^+^CD163^+^CD206^+^TLR4^+^ CD80^+^CD86^+^ (%leukocytes)	0.04 ± 0.08	0.002 ± 0.03	***p*** **= 0.036**	0.0006 ± 0.001	**< 0.0001**
CD204^+^CD163^+^CD206^+^TLR4^+^ CD80^+^CD86^+^ (%CD204^+^)	1 ± 5.6	0.39 ± 4	***p*** **= 0.021**	0.08 ± 0.2	**< 0.0001**

By Mann-Whitney test, several mixed M1/M2 cell populations were found to show significantly higher percentages (p MW highlighted in bold) in SSc patients affected by ILD, compared to SSc patients with no ILD. On the right, Kruskal-Wallis test was performed adding HSs data, obtaining more significant results (p KW)

No significant difference was reported for total CD204^+^ cell percentage, over circulating leukocytes, between SSc-ILD patient and SSc-No ILD patient groups (Table 2).

Considering the CD204^+^ cell population, SSc-ILD patients showed a significant increased percentage of circulating CD204^+^CD163^+^ cells compared to SSc-No ILD patients (Table 2). Likewise, circulating CD204^+^CD163^+^TLR4^+^ cells, CD204^+^CD163^+^CD206^+^TLR4^+^ cells, showed significant higher percentages in the SSc-ILD group (Table 2). Among CD204^+^CD163^+^TLR4^+^CD206^+^ cells, only CD14^−^ and not CD14^+^ cells showed significantly higher percentages in the SSc-ILD group (Table 2).

**Fig. 2:**
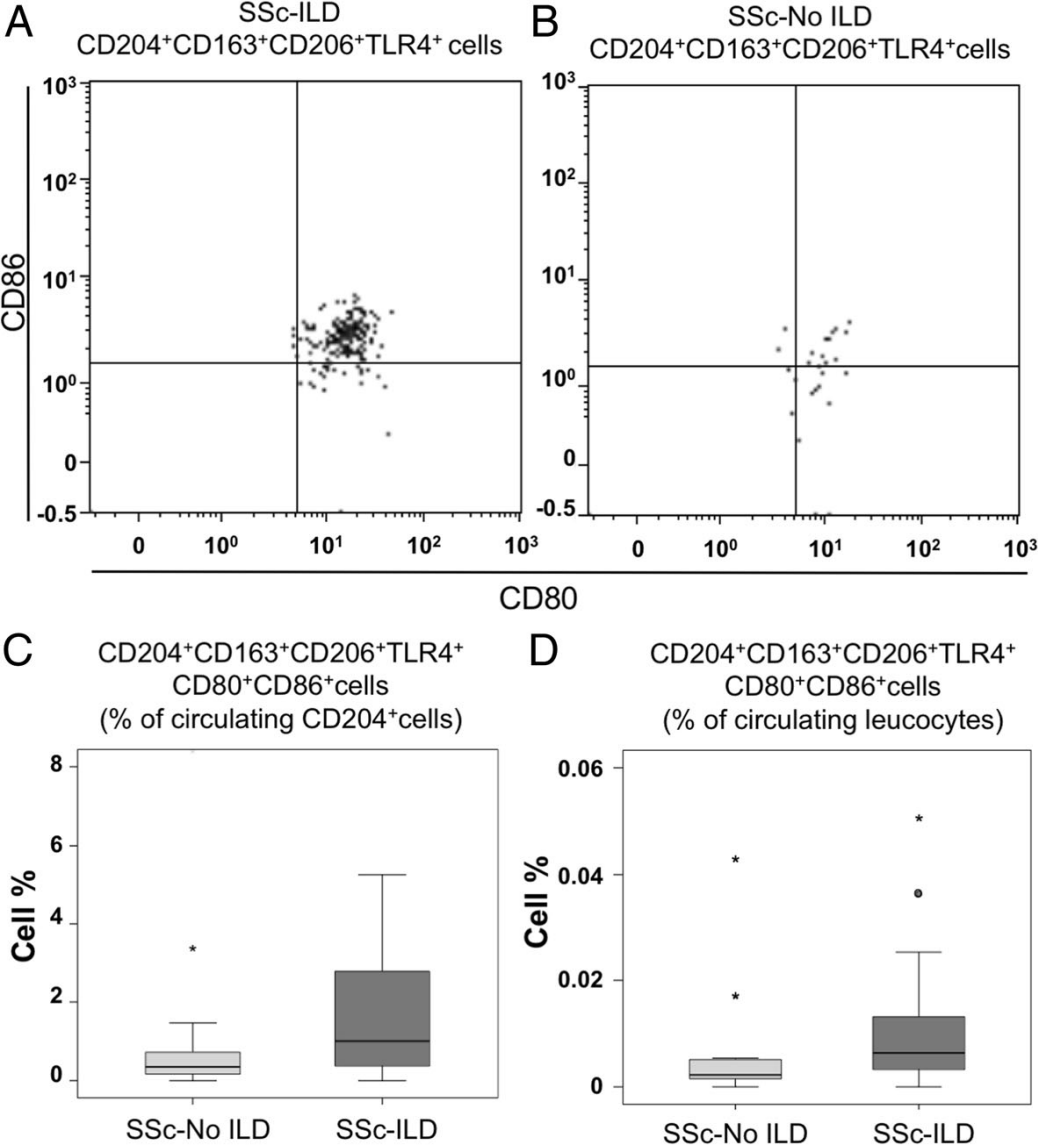
ILD affected SSc patients: associations with mixed M1 M2 cells percentage. **a** and **b**, representative dot plots from the flow cytometry analysis of the CD204 + CD163 + CD206 + TLR4 + CD80 + CD86+ cell subset in SSc patients affected by ILD and not affected by ILD are shown. Mixed M1/M2 cells expressing CD80 and CD86 markers, among CD204^+^CD163^+^TLR4^+^CD206^+^ cells, resulted significantly increased in percentage in the SSc-ILD group compared to the SSc-No ILD group, if calculated both over total CD204^+^ cells (**c**) and over total circulating leukocytes (**d**)

Remarkably, in the CD204^+^CD163^+^CD206^+^TLR4^+^ cell population, mixed M1/M2 phenotype cells expressing CD80 and CD86 markers resulted significantly increased in the SSc-ILD group compared to the SSc-No ILD group (Fig. 2 a, b, c, d and Table 2).

No differences were observed between SSc-ILD and SSc-No ILD patients in the percentage of total circulating CD14^+^ cells (6.68 ± 1.8% and 7.57 ± 2.5%, respectively, *p* = 0.18).

No differences were observed between SSc-ILD and SSc-No ILD patients in the percentage of circulating monocytes/macrophages expressing only surface markers considered to be M1 specific.

### Monocytes/macrophages phenotype and single CT scan alterations associated with interstitial lung disease, in SSc patients

Interestingly, SSc patients showing fibrotic changes diffused to upper and lower lobes at lung CT scan, seemed to be characterized by a slightly higher percentage of mixed M1/M2 monocytes/macrophages and characterized as CD14^+^CD206^+^CD163^+^CD80^+^CD86^+^ compared to patients with less or no lung fibrosis (0.001 ± 0.008 and 0.0006 ± 0.006, *p* = 0.044). Coherently, the same cell population showed higher percentages in patients presenting bronchiectasis or bronchiolectasis (0.002 ± 0.008% and 0.0006 ± 0.006%, *p* = 0.021).

No significant difference was reported in circulating monocyte/macrophage phenotype between patients with reported ground glass opacities localized at lower or upper lung lobes or diffused to both locations at lung CT scan. Similarly, no significant difference was observed in SSc patients for whom peripheral septal thickening, apical fibrotic changes, or enlarged mediastinal nodes were reported at lung CT scan.

### Correlations between circulating monocyte/macrophage phenotype, PFTs and sPAP values, in SSc patients

**Fig. 3:**
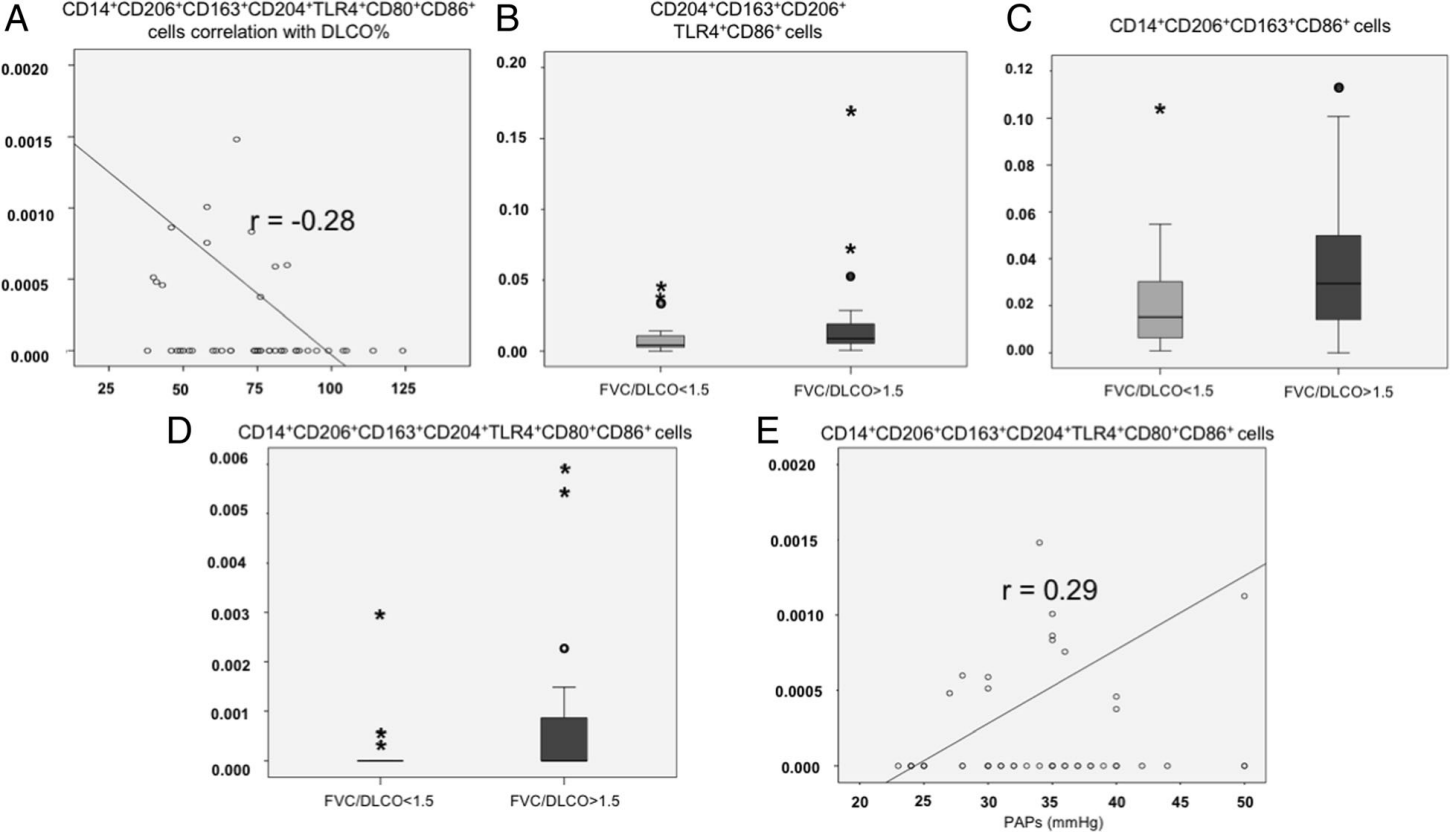
DLCO%, FVC/DLCO, sPAP values associations with mixed M1 M2 phenotype cells percentages. **a** a linear correlation between the mixed M1/M2 phenotype subset CD14^+^CD206^+^CD163^+^CD204^+^TLR4^+^CD80^+^CD86^+^ cell percentages and DLCO% values is shown. **b** an FVC/DLCO ratio higher than 1.5 resulted associated with CD204^+^CD163^+^CD206^+^TLR4^+^CD86^+^ cell subset percentage. **c** an FVC/DLCO ratio higher than 1.5 resulted associated with CD14^+^CD206^+^CD163^+^CD86^+^ cell subset percentage. **d** an FVC/DLCO ratio higher than 1.5 resulted associated with CD14^+^CD206^+^CD163^+^CD204^+^TLR4^+^CD80^+^CD86^+^ cell subset. **e** a linear correlation between the mixed M1/M2 phenotype subset CD14^+^CD206^+^CD163^+^CD204^+^TLR4^+^CD80^+^CD86^+^ cell percentages and sPAP values is shown

Higher percentages of circulating mixed M1/M2 monocyte/macrophage subset, characterized as CD14^+^CD206^+^CD163^+^CD204^+^TLR4^+^CD80^+^CD86^+^ cells, showed a weak linear negative correlation with DLCO% (*p* = 0.046, *r* = − 0.28, Fig. 3a).

No linear correlations were observed between macrophage subsets phenotype and FVC% values.

A FVC/DLCO ratio higher than 1.5 was associated with several circulating monocyte/macrophage subset percentages, and in particular: CD204^+^CD163^+^ cells calculated over total CD204^+^ cells (FVC/DLCO< 1.5 = 9 ± 15% vs. FVC/DLCO> 1.5 = 13 ± 14%, *p* = 0.006), CD204^+^CD163^+^TLR4^+^ cells calculated over CD204^+^ cells (FVC/DLCO< 1.5 = 3 ± 15% vs. FVC/DLCO> 1.5 = 6.4 ± 15%, *p* = 0.025), CD204^+^CD163^+^TLR4^+^ cells calculated over total leucocytes (FVC/DLCO< 1.5 = 0.02 ± 0.25%, vs. FVC/DLCO> 1.5 = 0.04 ± 0.02%, *p* = 0.039), CD204^+^CD163^+^CD206^+^TLR4^+^CD86^+^ cells calculated over total leucocytes (FVC/DLCO< 1.5 = 0.008 ± 0.03 vs. FVC/DLCO> 1.5 = 0.04 ± 0.09% %, *p* = 0.041, Fig. 3b). As regards cell subsets calculated over total CD14^+^ cells, significant differences were observed with an FVC/DLCO ratio lower or higher than 1.5: CD14^+^CD163^+^ cells (FVC/DLCO< 1.5 = 5.8 ± 2.4% vs. FVC/DLCO> 1.5 = 6.9 ± 2.3%, *p* = 0.044), CD14^+^CD206^+^ cells (FVC/DLCO< 1.5 = 6.1 ± 2.8% vs. FVC/DLCO> 1.5 = 7.2 ± 2.8%, *p* = 0.05), CD14^+^CD206^+^CD163^+^ cells (FVC/DLCO< 1.5 = 5.9 ± 2.7% vs. FVC/DLCO> 1.5 = 6.8 ± 2.7%, *p* = 0.046), CD14^+^CD206^+^CD163^+^CD86^+^ cells (FVC/DLCO< 1.5 = 0.01 ± 0.022% vs. FVC/DLCO> 1.5 = 0.03 ± 0.03%, *p* = 0.034, Fig. 3c), and CD14^+^CD206^+^CD163^+^CD204^+^TLR4^+^CD80^+^CD86^+^ cells (FVC/DLCO< 1.5 = 0.0001 ± 0.0005% vs. FVC/DLCO> 1.5 = 0.001 ± 0.005%; *p* = 0.005, Fig. 3d, Additional file 3). Moreover, the higher percentage of mixed M1/M2 CD14^+^CD206^+^CD163^+^CD204^+^TLR4^+^CD80^+^CD86^+^ cell subset correlated positively with the sPAP value (*p* = 0.028, *r* = 0.29, Fig. 3e).

## Discussion

The results of the present study demonstrated that a population of circulating cells belonging to the monocyte/macrophage lineage and expressing surface markers of both M1 and M2 phenotypes exists, in significantly high percentages, in SSc patients diagnosed as affected by ILD at CT scan. Additionally, higher percentages of mixed M1/M2 circulating monocytes/macrophages resulted linearly correlated with lower values of DLCO%, with an FVC/DLCO ratio higher then 1.5, and with higher PAPs values. Finally, mixed M1/M2 cell populations demonstrated to be associated with positivity for Scl-70 antibody, a well-known predictor for lung function decline, and less strictly with diffused disease form [29, 30].

Of note, among the circulating leucocyte population, two initial gating strategies moving from CD204^+^ cells and CD14^+^ cells gave significant results in patients affected by SSc-related ILD. On the contrary, no significant results were obtained when investigating CD80^+^CD86^+^ cells as initial gating strategy, thus confirming previous studies demonstrating a prevalent presence of markers characteristically linked with the alternately activated macrophage phenotype in SSc patients [31, 32].

The cellular subsets that appear to be correlated with pulmonary involvement seem to be essentially two: the first characterized by the positivity for CD204 and other M2 and M1 surface markers but negative for CD14; the second subset made up of cells positive for CD14 and for other M1 and M2 surface markers, possibly less mature than those positive for CD204. As described by Lambert et al., although the mature circulating monocytes are classically characterized by their expression of CD14, this marker seems not to be considered the hallmark for monocyte identification, in particular in the late phase of maturation, which is accompanied by the expression of CD16 [27]. Moreover, the CD14^low^CD16^+^ monocyte subset seems to correspond to M2 monocytes/macrophages. Interestingly, in a recent study by Lescoat et al., CD16^+^ monocytes resulted not only associated with pulmonary fibrosis, severity of the restrictive disease and reduction of DLCO but also they were described as precursors of M2 macrophages [33]. Accordingly, even if CD16 was not evaluated in our present study, it may be possible to speculate that the mixed M1/M2 population derived from CD204^+^ monocytes, which is CD14^−^ and associated with ILD, might be positive for CD16.

The presented findings could open to the perspective of a possible role of mixed M1/M2 cells in SSc and SSc-associated ILD pathogenesis, or at least as potential biomarkers for lung involvement in SSc. In a recent study, an integrated genomic approach using a consensus clustering was performed to compare the gene expression profiles of SSc biopsies from different tissues, including skin, lung and peripheral blood mononuclear cells. The authors described the concept of the immune-fibrotic axis, that is a common pathogenic gene expression signature indicative of the fundamental role of macrophages [34].

In particular, a distinct macrophage signature associated with the alternative activation was observed in SSc-associated pulmonary fibrosis and in the skin of patients with an “inflammatory” SSc gene expression signature, suggesting that there are subtle differences in the macrophage gene expression in lung and skin [34]. Based on these observations, the authors concluded that the plasticity of the monocyte/macrophage lineage is likely to be central to the divergence of fibrotic processes in different SSc-affected tissues and is an important component of an immune-fibrotic axis driving disease pathogenesis [34].

At the same time, the statistics from the present study show the constant presence, in patients affected or not by ILD, of a great variability of cell percentages obtained from the analysis of the various circulating monocyte/macrophages subsets, testified by large standard deviations, and reflecting considerable heterogeneity in cell size. Although this phenomenon could be attributed to the limited sample size, it may also be related to the cells phenotype plasticity in relation with different environmental stimuli and to their capacity to rapidly change accordingly, resulting in wide distribution of surface markers positivity at the time of the analysis [20].

Several authors have focused on how macrophage phenotype modifications could contribute to the development of lung fibrotic and neoplastic disorders [14, 16]. However, the studies often concentrated on the expression of single phenotype surface markers or soluble molecules and their possible association with diseases clinical features. It is the case, for example, of the presence of the M2 markers CD163 or soluble (s)CD163 that showed to be higher in presence of several organ involvements, like in ILD and pulmonary arterial hypertension (PAH), in SSc and other autoimmune diseases, like polymyositis and dermatomyositis [28, 29].

Serum and urinary sCD163 concentrations were very recently investigated as possible biomarkers in SSc patients compared to HSs and a study demonstrated that serum sCD163 levels were significantly higher in SSc patients compared to HSs [35]. However, sCD163 concentrations were not associated with clinical, laboratory, and instrumental characteristics of SSc patients [35].

Other authors described M2 macrophages in SSc patients and their association with several clinical parameters. Higher percentages of circulating cells positive for CD204, CD163, and CD14 were shown to correlate with skin involvement [31].

Circulating alternatively activated CD14^+^ macrophages, expressing high levels of CD206 were demonstrated to be associated with PAH [32].

A recent study investigated the phenotype of human alveolar macrophages (AMs) in adults living in UK and Malawi, demonstrating that the majority of AMs expressed high levels of M1 and M2 markers simultaneously. As the authors postulate, it is possible that in the healthy lung mucosa, combined M1/M2 features could confer to AMs the ability of maintaining a balance between immune tolerance and protective immunity. On the contrary, a similar circulating phenotype could exert a pathogenic role in SSc patients [21].

Moreover, it was demonstrated that monocyte-derived macrophages co-expressing CD206, CD163 and CD169 were significantly higher in SSc-ILD than in lung cancer or sarcoidosis and that a similar macrophage phenotype was obtained from the analysis of blood-monocytes derived macrophages in SSc patients [36].

However, to our knowledge, no one has so far attempted such a wide phenotype characterization of circulating monocytes/macrophages in connection with the development of SSc-related lung and right heart complications.

This study has several limits. First of all, the relatively small patient population. In fact, even if in a previous paper an effect of treatment regimens on circulating monocyte/macrophage phenotype was observed [22], in the present study a further subgroup analysis of patients with lung involvement and undergoing specific treatments compared to non-treated patients, seems not to be possible without losing too much statistical power. A follow study including a larger number of SSc patients is planned. Accidentally, most of the patients in the study population had consistently high levels of FVC (average values 104%) and very few patients had a severe restrictive disease, therefore it was not possible to ascertain the presence of a linear correlation with FVC% or with its decline.

Secondarily, the evaluation of only circulating cells is also a limitation. In fact, while interpreting the presented results, it should be kept in mind that a wide debate is taking place on the possible link between circulating and tissue resident monocyte/macrophage cells, questioning the dogma on the monocyte origin of tissue macrophages. Infiltrating macrophages, observed in the diseased tissue, seem to derive from circulating monocytes, but several studies opposed the monocyte origin for tissue resident cells [37, 38].

Moreover, emerging immunological theories attribute to organ and tissues the control of immune system activation and its forms (Th1 or Th2 like) [39]. Although the surface markers investigated in the study (primarily CD204, CD163 and CD206) are considered specific for the characterization of M2 polarized cells, they can also be expressed by dendritic cells [40, 41]. Therefore, based on the gating strategies proposed in our study, a percentage of dendritic cells might be present in the described circulating cell subsets. Monocytes, macrophages and dendritic cells are all members of the mononuclear phagocyte system which is involved in multiple functions during immune responses and, although these cells may be distinguished based on functional and phenotypical characteristics, some cell features are often overlapping and the distinction or classification is challenging [42]. The possible implication of dendritic cells in the development of SSc was recently highlighted in a recent paper by Silvan et al. describing that these cells expressing high levels of PSGL-1 were associated with the presence of interstitial lung disease in SSc patients [43].

Finally, the very low percentage of the newly described circulating cells could make it not easy to use them as a disease biomarker. Evidently, further and larger studies and possibly the sorting of the mixed M1/M2 population will be useful for the evaluation of the importance of this phenotype in both pathogenic, diagnostic or therapeutic perspectives.

Although the identification of these circulating mixed M1/M2 cells was performed through the evaluation of the most investigated and specific markers related to each polarization status and in a previous study they were found significantly increased in SSc patients compared to HSs, the analysis of the marker expression through the detection of the mean fluorescence intensity (MFI), not investigated in this study, might represent a further important aspect that may contribute to understand their possible role in SSc pathogenesis.

In accordance with our results on a possible circulating “scleroderma” macrophage with M1/M2 phenotype, very recently, Moreno-Moral et al. have found in 57 SSc patients, through RNA sequencing and genome-wide genotyping, a mixed macrophage activation signature, characterized by the downregulation of interferon gamma response, attesting for an M2 polarization, but also by the downregulation of the interleukin (IL)-6/JAK/STAT3 signalling pathway, suggesting for a restricted M2 activity [44]. The authors observe that the circulating monocyte/macrophage phenotype could contrast with macrophage signature in tissues such as lung, in which a STAT3-dependent expression of CD163 was associated with pulmonary fibrosis [45].

Interstitial lung disease is a major cause of morbidity and mortality in systemic sclerosis (SSc). Notwithstanding many authors concentrated their attention in this direction, the pathogenic mechanisms of SSc-related ILD remain unknown, and limited therapeutic effects are obtained with the available treatments [46–48].

The knowledge of the mechanisms that initiate the pathogenesis of pulmonary damage or of an easily evaluable biomarker associated with such involvement would be crucial.

Supported by the discovery of interferon (IFN) α, tumor necrosis factor (TNF) α, TLRs, transforming growth factor (TGF) β, platelet derived growth factor (PDGF), genes signatures in SSc, we hypothesized that both Th1 and Th2 activation signals could derive from different damaged tissues, determining the development of circulating mixed M1/M2 cells. At the same time, more polarized responses could possibly develop at tissues level, such as in lungs [39].

## Conclusions

In conclusion, it is possible to state that this is the first study showing an association of an M1/M2 monocyte/macrophage phenotype in SSc patients to SSc-related ILD functional and radiological data.

The evaluation of the existence of circulating mixed M1/M2 monocyte/macrophage phenotype and its clinical associations in SSc patients, should be considered as the first step towards a conclusion for a possible role as a pathogenic factor or as an early biomarker for organ involvement in SSc-related ILD. Such a phenotype could be found also in other pulmonary diseases, at the circulatory or tissue level. The presented acquisitions could therefore be considered as an opening to later studies on a wide phenotype characterization of macrophages at the level of different diseased tissues including lung, kidney, heart, and skin in SSc but also in other fibrotic disorders. Furthermore, isolation and functional study of the described cells are under evaluation for the remarkable values they could have for physiopathology, diagnostic and therapeutic purposes [49].

## Additional files

Additional file 1: Gating strategies for the detection of circulating M1, M2 and mixed M1/M2 cells in systemic sclerosis patients and healthy controls. (A) Representative flow cytometry scatter plot and scatter dot plot with median and interquartile range of the initial gating strategy starting from the circulating CD14^+^cells percentage (%) in the leucocyte population; (B) Representative flow cytometry panels with quadrant regions and scatter dot plot representation of the of circulating CD14^+^CD206^+^CD163^+^cells in the CD14^+^cell population; (C) CD14^+^CD206^+^CD163^+^CD204^+^TLR4^+^cells in the CD14^+^CD206^+^CD163^+^cell subset and (D) CD14^+^CD206^+^CD163^+^CD204^+^TLR4^+^CD80^+^CD86^+^cells in the CD14^+^CD206^+^CD163^+^TLR4^+^cell subset of healthy subjects (HSs) and systemic sclerosis patients (SSc pts). (E) Representative flow cytometry scatter plot and scatter dot plot with median and interquartile range of the initial gating strategy starting from the circulating CD204^+^cells percentage (%) in the leucocyte population; (F) Representative flow cytometry panels with quadrant regions and scatter dot plot representation of the of circulating CD204^+^CD163^+^CD206^+^cells in the CD204^+^cell population; (G) CD204^+^CD163^+^CD206^+^TLR4^+^cells in the CD204^+^CD163^+^CD206^+^cell subset; (H) CD204^+^CD163^+^CD206^+^TLR4^+^CD80^+^CD86^+^cells and (I) CD14^+^ and CD14^−^cells in the CD204^+^ 163^+^CD206^+^TLR4^+^cell subset of HSs and SSc pts. (J) Representative flow cytometry scatter plot of the initial gating strategy starting from the circulating CD80 + CD86 + cells percentage (%) in the leucocyte population and (L) representative flow cytometry panels with quadrant regions of the of circulating CD80^+^CD86^+^TLR2^+^TLR4^+^cells in the CD80^+^CD86^+^cell population of HSs and SSc pts. Statistical analysis was performed by Mann-Whitney non-parametric test and *p*-values lower than 0.05 was considered as statistically significant. (TIF 1646 kb)

Additional file 2: Differences in the percentage of mixed M1/M2 cells in systemic sclerosis patients with or without Ab anti Scl70 positivity and healthy subjects. Cell populations with a mixed M1/M2 phenotype, showing significantly different percentages between Scl70 antibody positive (Scl70 + Pts) and Scl70 antibody negative (Scl70-Pts) patients at Mann-Whitney were then analyzed together with those from age and gender matched healthy subjects (HSs) through Kruskal-Wallis test. HSs showed constantly lower percentages compared to Scl70 + Pts and Scl70-Pts. (TIF 514 kb)

Additional file 3: Differences in the percentage of M2 and mixed M1/M2 cells in systemic sclerosis patients with an FVC/DLCO ratio lower or higher than 1.5. With both gating strategies, one based on CD204 positivity and one based on CD14 positivity, cell populations with an M2 or a mixed M1/M2 phenotype, showed significantly higher percentages in patients with an FVC/DLCO ratio higher then 1.5 compared to patients with an FVC/DLCO ratio lower than 1.5. (DOCX 13 kb)

## Abbreviations

Ab anti-Scl70: anti-topoisomerase antibody
ACA: Anti-centromere antibodies
ACR: American College of Rheumatology
ANA: Anti-nuclear antibody
CT: Computed tomography;
dcSSc: Diffused cutaneous systemic sclerosis
DLCO: Diffusing capacity of the lungs for carbon monoxide
EULAR: European League Against Rheumatism
FVC: Forced vital capacity
IL: Interleukin
ILD: Interstitial lung disease
lcSSc: Limited cutaneous systemic sclerosis
MRC1: Gene codifying for c-type mannose receptor 1
NSIP: Non-specific interstitial pneumonia
PFTs: Pulmonary function tests
pro-BNP: Pro-hormone of brain natriuretic peptide
PTs: Patients
RP: Raynaud’s phenomenon
SD: Standard deviation
sPAP: Systolic pulmonary artery pressure
SSc: Systemic sclerosis
Th: T helper
TLR: Toll-like receptors
