# Supplementary figures and images for: A circulating cell population showing both M1 and M2 monocyte/macrophage surface markers characterizes systemic sclerosis patients with lung involvement
Source: Respir Res. 2018 Sep 24;19:186. doi: 10.1186/s12931-018-0891-z (PMC6154930; doi:10.1186/s12931-018-0891-z)

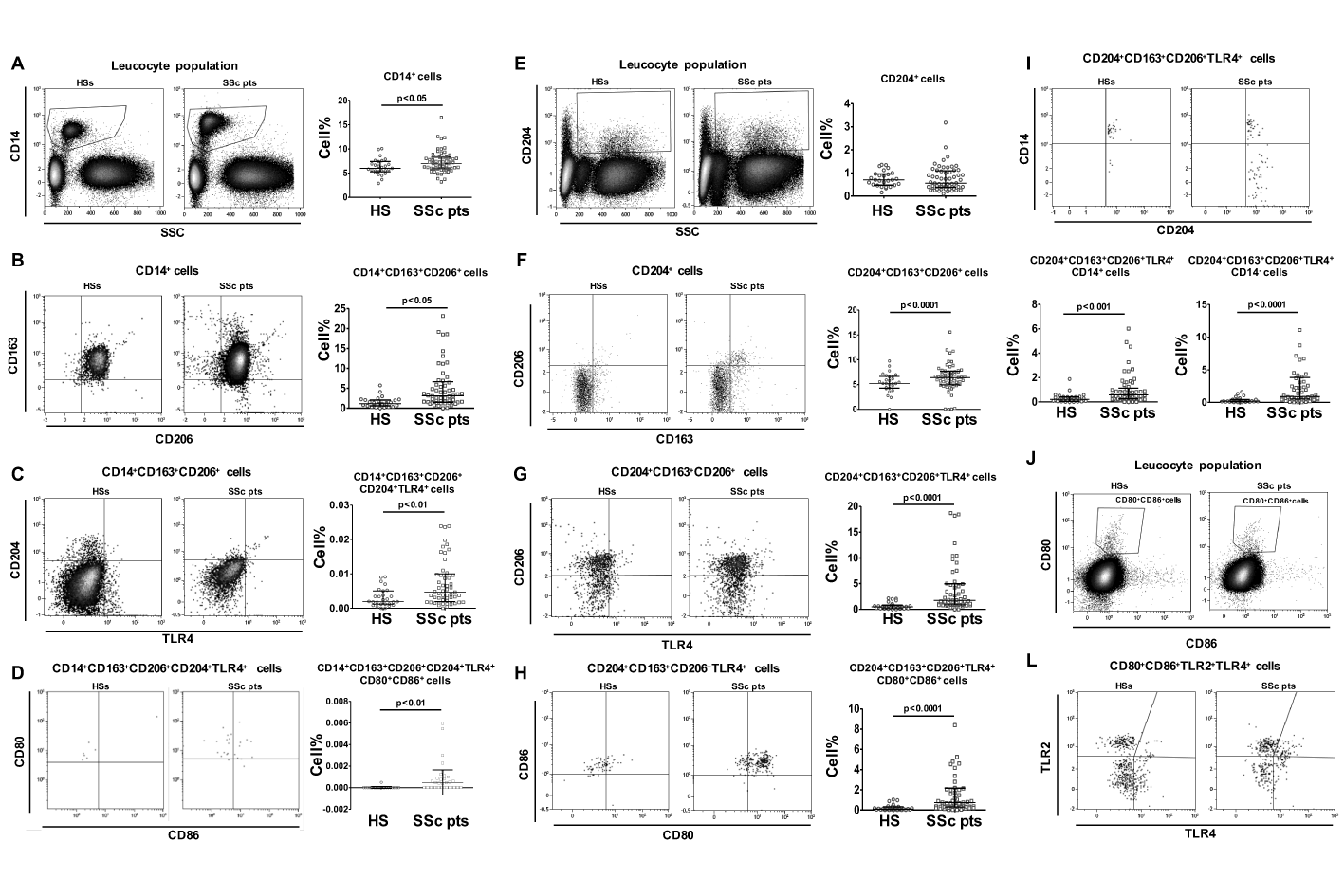

Supplement: Supplementary file 1 — Gating strategies for the detection of circulating M1, M2 and mixed M1/M2 cells in systemic sclerosis patients and healthy controls. (A) Representative flow cytometry scatter plot and scatter dot plot with median and interquartile range of the initial gating strategy starting from the circulating CD14+cells percentage (%) in the leucocyte population; (B) Representative flow cytometry panels with quadrant regions and scatter dot plot representation of the of circulating CD14+CD206+CD163+cells in the CD14+cell population; (C) CD14+CD206+CD163+CD204+TLR4+cells in the CD14+CD206+CD163+cell subset and (D) CD14+CD206+CD163+CD204+TLR4+CD80+CD86+cells in the CD14+CD206+CD163+TLR4+cell subset of healthy subjects (HSs) and systemic sclerosis patients (SSc pts). (E) Representative flow cytometry scatter plot and scatter dot plot with median and interquartile range of the initial gating strategy starting from the circulating CD204+cells percentage (%) in the leucocyte population; (F) Representative flow cytometry panels with quadrant regions and scatter dot plot representation of the of circulating CD204+CD163+CD206+cells in the CD204+cell population; (G) CD204+CD163+CD206+TLR4+cells in the CD204+CD163+CD206+cell subset; (H) CD204+CD163+CD206+TLR4+CD80+CD86+cells and (I) CD14+ and CD14−cells in the CD204+ 163+CD206+TLR4+cell subset of HSs and SSc pts. (J) Representative flow cytometry scatter plot of the initial gating strategy starting from the circulating CD80 + CD86 + cells percentage (%) in the leucocyte population and (L) representative flow cytometry panels with quadrant regions of the of circulating CD80+CD86+TLR2+TLR4+cells in the CD80+CD86+cell population of HSs and SSc pts. Statistical analysis was performed by Mann-Whitney non-parametric test and p-values lower than 0.05 was considered as statistically significant. (TIF 1646 kb) [file 12931_2018_891_MOESM1_ESM.tif]

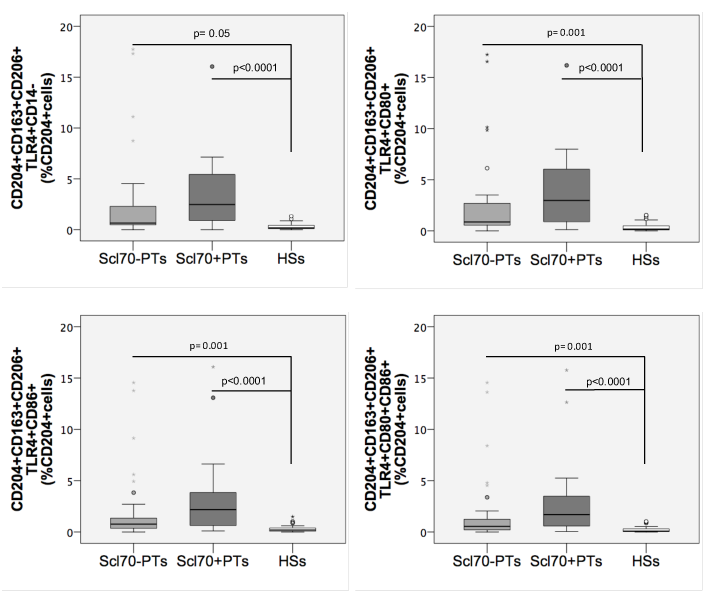

Supplement: Supplementary file 2 — Differences in the percentage of mixed M1/M2 cells in systemic sclerosis patients with or without Ab anti Scl70 positivity and healthy subjects. Cell populations with a mixed M1/M2 phenotype, showing significantly different percentages between Scl70 antibody positive (Scl70 + Pts) and Scl70 antibody negative (Scl70-Pts) patients at Mann-Whitney were then analyzed together with those from age and gender matched healthy subjects (HSs) through Kruskal-Wallis test. HSs showed constantly lower percentages compared to Scl70 + Pts and Scl70-Pts. (TIF 514 kb) [file 12931_2018_891_MOESM2_ESM.tif]
