# Supplementary material for: A circulating cell population showing both M1 and M2 monocyte/macrophage surface markers characterizes systemic sclerosis patients with lung involvement
Source: Respir Res. 2018 Sep 24;19:186. doi: 10.1186/s12931-018-0891-z (PMC6154930; doi:10.1186/s12931-018-0891-z)
Supplement: Supplementary file 3 — Differences in the percentage of M2 and mixed M1/M2 cells in systemic sclerosis patients with an FVC/DLCO ratio lower or higher than 1.5. With both gating strategies, one based on CD204 positivity and one based on CD14 positivity, cell populations with an M2 or a mixed M1/M2 phenotype, showed significantly higher percentages in patients with an FVC/DLCO ratio higher then 1.5 compared to patients with an FVC/DLCO ratio lower than 1.5. (DOCX 13 kb) [file 12931_2018_891_MOESM3_ESM.docx]

**Additional File 3**

| **ANALYSIS OF CIRCULATING CELLS** | FVC/DLCO<1.5 | FVC/DLCO>1.5 | **p** |
| --- | --- | --- | --- |
| **CD204^+^CD163^+^ (%CD204^+^)** | 9±15 | 13±14 | p=0.006 |
| **CD204^+^CD163^+^TLR4^+^ (%leukocytes)** | 0.02 ±0.25 | 0.04±0.02 | p=0.34 |
| **CD204^+^CD163^+^TLR4^+^(%CD204^+^)** | 3±15 | 6.4±15 | p=0.025 |
| **CD204^+^CD163^+^CD206^+^TLR4^+^CD86^+^**  **(%leukocytes)** | 0.008 ± 0.03 | 0.04± 0.09 | p=0.082 |
| **CD14^+^CD163^+^** | 5.8±2.4 | 6.9±2.3 | 0.044 |
| **CD14^+^CD206^+^** | 6.1±2.8 | 7.2±2.8 | 0.05 |
| **CD14^+^CD206^+^CD163^+^** | 5.9±2.7 | 6.8±2.7 | 0.046 |
| **CD14^+^CD206^+^CD163^+^CD86^+^** | 0.01±0.022 | 0.03±0.03 | 0.034 |
| **CD14^+^CD206^+^CD163^+^CD204^+^TLR4^+^CD80^+^CD86^+^** | 0.0001±0.0005 | 0.001±0.005 | p=0.005 |
